# Psoriatic Dactylitis: Current Perspectives and New Insights in Ultrasonography and Magnetic Resonance Imaging

**DOI:** 10.3390/jcm10122604

**Published:** 2021-06-12

**Authors:** Nicolò Girolimetto, Ivan Giovannini, Gloria Crepaldi, Gabriele De Marco, Ilaria Tinazzi, Niccolò Possemato, Pierluigi Macchioni, Rebecca McConnell, Dennis McGonagle, Annamaria Iagnocco, Alen Zabotti

**Affiliations:** 1 Department of Rheumatology, Azienda USL-IRCCS di Reggio Emilia, 42122 Reggio Emilia, Italy; Niccolò.Possemato@asmn.re.it (N.P.); pierluigi.macchioni@ausl.re.it (P.M.); 2 Department of Medical and Biological Sciences, Institute of Rheumatology, University Hospital ‘Santa Maria della Misericordia’, 33100 Udine, Italy; i.giovannini.qwerty@gmail.com (I.G.); zabottialen@gmail.com (A.Z.); 3 SSD Reumatologia, AO Ordine Mauriziano di Torino, 10128 Turin, Italy; gloria.crepaldi@gmail.com; 4 NIHR Leeds Biomedical Research Centre, Leeds Institute of Rheumatic and Musculoskeletal Medicine, University of Leeds, Chapel Allerton Hospital, Leeds LS7 4SA, UK; G.DeMarco@leeds.ac.uk (G.D.M.); D.G.McGonagle@leeds.ac.uk (D.M.); 5 Unit of Rheumatology, IRCSS Ospedale Sacro Cuore Don Calabria, 27024 Negrar di Valpolicella, Italy; ilariatinazzi@yahoo.it; 6 Department of Surgical Sciences, Università degli Studi di Torino, 10124 Turin, Italy; 2rebeccamcconnell@gmail.com; 7 Academic Rheumatology Centre, Dipartimento di Scienze Cliniche e Biologiche, Università degli Studi di Torino, 10043 Turin, Italy; annamaria.iagnocco1@gmail.com

**Keywords:** dactylitis, psoriatic arthritis, ultrasound, imaging, score

## Abstract

Dactylitis, one of the most typical features of psoriatic arthritis (PsA), is the diffuse swelling of the digits and is determined by the involvement of different anatomic structures, including: the subcutaneous fibrous tissue “accessory pulley” system; flexor tendons, with their related structures; the articular synovium; the small enthesis of the hands. Dactylitis is currently considered both a marker of disease activity and severe prognosis and its importance in PsA is emphasized by the inclusion in the classification criteria of PsA. This review focuses on the role of imaging in the management of PsA patients with dactylitis in clinical practice and in a research setting. Furthermore, imaging could be a valuable tool to assist in unravelling some of the underlying mechanisms of the onset and chronicization of dactylitis in PsA patients.

## 1. Introduction

**Figure 1:**
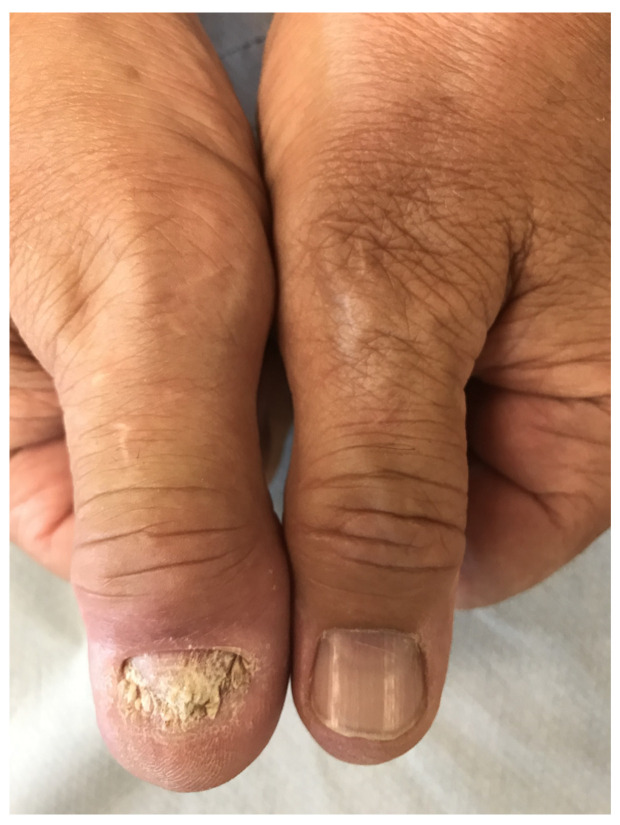
Photograph of hand dactylitis: The first finger of the right hand is affected by dactylitis. Image courtesy of Dr. Alen Zabotti.

Dactylitis originates from the Greek word δακτύλος, meaning finger, and is the diffuse swelling of an entire digit typically related to an underlying inflammation or an infiltrative disorder (Figure 1) [1,2]. Psoriatic arthritis (PsA) is the most common disease that causes dactylitis due to joint, tendon, and soft tissue inflammation. The resulting “sausage digit” can cause uniform finger enlargement so extensive that the joint swelling may be indistinguishable from the swelling of the surrounding tissues [3].

Forty-eight percent of PsA patients experience dactylitis at some point during their disease [4], and its importance in the spondylarthritis assessment is supported by its inclusion in two influential diagnostic guides: the ClASsification for Psoriatic ARthritis (CASPAR) criteria [5] and the Assessment of SpondyloArthritis International Society (ASAS) criteria [6].

Clinically, dactylitis can occur with an acute or chronic appearance. Acute/tender dactylitis, is characterized by a slightly reddish, tender, and swollen digit. Chronic dactylitis, also called “cold” dactylitis, presents as a non-tender, swollen finger. It is unknown whether a lack of tenderness represents the resolution of an acute dactylitis or suggests inactive disease [7]. Recent studies suggest that the absence of tenderness might be explained by the anatomical compartmentalization of dactylitic-related sonographic abnormalities [8,9].

The clinical relevance of dactylitis should not be undervalued; dactylitis is related to impaired basic motor function and seems to have prognostic value in the affected finger, indicating general disease severity/gravity, and acting as a measure of PsA progression [4]. Brockbank et al. reported on the radiological damage found more frequently in digits with dactylitis. Dactylitis commonly presents in an asymmetrical distribution and is more frequent in the feet than in the hands [4]. When hands are involved, the most frequently affected digits are the second and third digit (index and middle finger) of the dominant hand, while the fourth toe is the most common foot involvement (usually bilaterally) [4]. As reported by McGonagle et al., the greater involvement of the feet over the hands and the association with the dominant hand may indicate that trauma or injury might play a role in triggering the disease [10], supporting the deep Koebner phenomenon theory [11]. The deep Koebner phenomenon is a skeletal variant of the cutaneous Koebner phenomenon, where new lesions occur in areas exposed to local biomechanical trauma in patients genetically predisposed to PsA [12,13]. In addition to local dactylitic or cutaneous lesions, previous reports also correlate biomechanical stress, such as physical trauma, to PsA development [14].

Ultrasound (US) and magnetic resonance imaging (MRI) indicated that dactylitis is a heterogeneous expression of psoriatic disease. Imaging studies of dactylitis characterized the association between the inflammatory involvement of tendons (such as flexor and extensor tendons), enthesitis, soft tissue thickening/oedema, and synovitis of metacarpophalangeal (MCP), proximal interphalangeal (PIP) and distal interphalangeal (DIP) joints [15,16].

## 2. Pathophysiology

Evidence that dactylitis occurs more frequently in the feet [4] and the second digit of the dominant hand points to the role of biomechanical factors such as physical “microtraumas”—repeated low-intensity injuries—as potential triggers for lesion development [11]. As first suggested by Moll and Wright in the 1970s [17], the deep Koebner phenomenon is a theoretical framework explaining individual lesion development from these biomechanical injuries across the spectrum of spondyloarthritis (SpA), including dactylitis. Indeed, thickening of the constraining flexor tendon accessory pulleys in dactylitis, which are susceptible to biomechanical stress, continues to support this phenomenon [11]. The flexor accessory pulleys also exhibit hypervascularization on US scans in acute dactylitis, demonstrating that biomechanical stresses can be important even in the earliest stages of dactylitis [18]. Recent enhancements in US and MRI resolution capacity are overcoming the traditional difficulty in visualizing pulleys, entheses, and peritendinous soft tissues [19], hence allowing the detection of inflammatory features in and around these small structures [20].

Despite imaging-driven progress in the understanding of dactylitis, immunopathological research into these lesions remains substantially underdeveloped due to significant limitations in the harvesting of appropriate human samples [10]. Animal models of SpA have partially overcome the lack of availability of histologic materials from the musculoskeletal structures of human digits with murine models of dactylitis. However, joints in rodents’ digits have closer anatomical proximity and lack the exact phenotypical replication of the clinical features in human SpA. In the DBA/1 model, mice developed dactylitis spontaneously under the influence of environmental factors such as cage size, and the related lesions displayed notable subcutaneous oedema and neutrophilic infiltrates [21,22]. Similar histological findings were also noted in the K5.Stat3C transgenic mice [23]. In the PSTPIP2 model, mice spontaneously developed swelling of the digits, alterations of the nails, and eventual osteolysis in the involved paws [24]. Lesions showed prominent macrophage infiltrates linked to the dysregulation of the innate immune system [24]. Systemic overexpression of interleukin(IL)-23, induced by the genetic manipulation of B10.III mice, led to remarkable swelling of the digits, pointing to a role of IL-23 in dactylitis [25]. The potential function of the IL-17/IL-23 axis in dactylitis is also highlighted by the SKG model [26,27], where mice affected by a mutation of the protein ZAP70 had hyper-functioning T cells; half of these mice developed dactylitis after immunization with curdlan. In the B10Q.Ncf1m1j/m1j model, mice developed enthesitis and dactylitis three days after an intra-peritoneal injection of mannan; the disease drivers were tumor necrosis factor (TNF)-producing myeloid cells and IL-17A-producing γδ T cells [28].

Immunogenetic studies in humans discovered the association of the alleles HLAB*2705 and HLAB*0801 with dactylitis, perhaps pointing to a prominent role of CD8+ T cells in dactylitis pathogenesis.

## 3. Imaging of Hand Dactylitis

Ultrasound and MRI studies suggest that flexor tenosynovitis is the major contributor to the physical appearance of dactylitis [29,30,31], while joint synovitis plays a minor role [32]. The diffuse circumferential thickening of the digit during the course of dactylitis seems to be mainly related to the inflammation of the digital entheseal structures, including the fibrous skeleton of the digit [33,34], resulting in an extensive digital soft-tissue oedema (STOe) and in the flexor tenosynovitis [15,19,33,35].

In 1996, Olivieri et al. [29] used MRI and US to establish the role of tenosynovitis and joint synovitis in producing the “sausage-like’’ digits; they found that both techniques showed synovial sheath enlargement due to continuous fluid collection. Later, MRI studies reported similar results of finger flexor tenosynovitis extending continuously into the palm of the hand [36,37]. An US study by Kane et al. [32] found flexor tenosynovitis in 96% of dactylitic digits, while also showing nodular tendon sheath thickening and sheath enlargement in both anteroposterior and transverse diameter with loss of normal tendon fibrillar pattern. Recent cross-sectional US studies involving only hand dactylitis reported a prevalence of flexor tenosynovitis ranging from 83 to 94% in grey scale (GS) and from 69 to 78% in power Doppler (PD) modes [8,9,38,39].

STOe was first described as a particular lesion of dactylitis in a 2006 sonographic study. They termed this subcutaneous and extratendinous digital soft tissue inflammation in dactylitic patients as “pseudo-tenosynovitis” [33].

Subsequent studies reported that STOe could be useful in the differentiation of early and established forms of PsA and rheumatoid arthritis (RA) [40,41]. Recently, STOe was sonographically defined as “abnormal hypoechoic/anechoic areas, diffused or localized within the subcutaneous tissue between the epidermis and the tendon-related anatomic structures, with local thickening, with or without local abnormal Doppler signal, visualized in two perpendicular planes, and not present on the contralateral side” [42,43]. The comparison with a digit not affected by the disease (contralateral or adjacent) is a fundamental step in the sonographic evaluation of STOe since subcutaneous tissue thickness is highly variable and dependent on an individual’s baseline [44]. Studies from our group reported that STOe was present in 75–91% of hand dactylitis (and associated with PD signal in 69–82% of cases) [8,9,38,39].

Synovitis may be present in about half of dactylitic digits, and the US features included symmetric and asymmetric hypoechoic synovial proliferation and symmetric hypoechoic joint effusion. On plain radiography, joint space narrowing and periostitis without erosions were significantly more frequent in dactylitic fingers [32]. In recent sonographic studies, GS synovitis involving at least one joint was observed in 40–43% of cases: 28–32% at the PIP joint; 14–15% at the MCP joint; 9–12% at the DIP joint. PD synovitis involving at least one joint was evident in 18–26% of cases [8,9,38,39]. PD synovitis was also most prevalent at the PIP joint (17–21%), followed by PD synovitis at the MCP and DIP joints (6–7% and 4–6%, respectively). Correspondingly, MRI also demonstrated a greater degree of synovitis severity in the PIP joints compared to the DIP joints [45]. Extensor tendon involvement was observed in 10–13% of dactylitic [8,9,38,39].

Flexor tendon pulleys are small, functional entheses that prevent tendons from bowstringing during finger flexion. Recent MRI and US data revealed the inflammatory involvement of these structures in PsA dactylitis as a likely part of the deep Koebner phenomenon [11,18,19]. In particular, thicker A1 pulleys were observed in PsA digits affected by dactylitis [11]. Hot dactylitis, known for its extracapsular inflammation, also exhibited abnormal increased PD signal of the accessory pulleys [18].

## 4. Imaging of Foot Dactylitis

Dactylitis involves the toes more frequently than the fingers [4]. A study of 260 PsA patients with dactylitis showed that approximately 66% had it only in the feet [4]. The clinical examination is often a sufficient method to diagnose toe dactylitis. Clinical examination showed 100% sensitivity and specificity compared to MRI for the diagnosis of flexor tenosynovitis in patients with toe dactylitis [46].

Many studies include both toes and fingers, and only a few studies exclusively examine the toes [35,47]. Two MRI studies on toe dactylitis suggested, as for hands, that flexor tenosynovitis is primarily responsible for the sausage-like phenotype, while joint synovitis is not required. An MRI study on 12 dactylitic toes from 10 SpA patients reported that flexor tenosynovitis was present in all cases. Joint effusion involving at least one joint was seen in 67% of the toes, while peritendinous soft tissue oedema was present in 92% [35]. Another study on 12 toes from 7 PsA patients had recorded a similarly high prevalence of tenosynovitis, while joint effusion was only present in a few cases [47]. A recent US study involving 31 PsA patients (17 toes and 12 fingers) showed a high prevalence of soft tissue thickening (81%) and subcutaneous edema (74%) [48]. GS synovitis and GS flexor tenosynovitis were present in 56–68% and 52% of patients, respectively. It is important to emphasize that the study did not use a high-frequency probe (only 6–15 MHz); moreover, chronic microtraumas could cause local edema more frequently in the foot than in the hand.

## 5. How to Score PsA Dactylitis

### 5.1. Clinical Scoring of PsA Dactylitis

Different methods can be used for scoring dactylitis [30]. A common and feasible method is to count the number of affected fingers during the clinical examination [30]. The Leeds Dactylitis Index (LDI) is another score based on the assessment of tenderness and digital circumference, evaluating the ratio of the affected digit to an unaffected digit on the opposite hand or foot. The finger circumference is usually assessed by a standard tool, called circumferometer [49]. Tenderness of the fingers and toes is based on the Ritchie index (graded 0–3), or, for the LDI-basic (LDI-b), tenderness is a dichotomic score (0 for non-tender, 1 for tender) [49,50]. LDI and LDI-b results are particularly suitable in clinical trials as an outcome measure. However, one of the problems with the LDI scoring system is its inability to evaluate dactylitis in its chronic, non-tender form.

The characterization of dactylitis features with US and MRI helps differentiate the underlying mechanisms and involved structures of acute (tender) and chronic (non-tender) presentations [38].

### 5.2. Ultrasound Scoring of PsA Dactylitis

Ultrasound detects multiple abnormalities in PsA dactylitis, and a meticulous standardized approach and an appropriate scanning technique are crucial for their assessment [42,51,52,53].

Recently, a dactylitis global sonographic score (DACTOS) was developed to evaluate the severity of dactylitis in clinical trials and clinical practice [43]. The DACTOS consists of a composite score for each elementary lesion of hand dactylitis: peritendon extensor inflammation (PTI) evaluated in B-mode and PD at the MCP and PIP joint levels (maximum score 4); STOe; flexor tenosynovitis evaluated in B-mode and PD in the most severely affected area of the digit (maximum score of 6 for each); EULAR-OMERACT combined score for synovitis evaluated at the MCP, PIP, and DIP joints (maximum score of 9). The sum of scores for all lesions ranges from 0 to 25 points for each involved finger. The DACTOS score is able to discriminate between dactylitic and normal fingers, is sensitive to change, and correlates with clinical parameters (VAS for pain and functional impairment and LDI-b values), making it an excellent tool for assessing the response to treatment [54]. However, it does not include small digital entheses. Signs of enthesitis, though common in dactylitis, present less frequently than other lesions [48]. The reliability of scoring enthesitis of larger entheses has been established in spondyloarthritis [55], but this is not validated for the small digital entheses and needs further exploration.

### 5.3. MRI Scoring of PsA Dactylitis

MRI is useful for quantifying the extent of inflammatory changes in dactylitic digits, and an appropriate scoring system has been proposed for use in clinical trials [38,45]. This score records each MRI-identified dactylitic feature as present or absent at the MCP, PIP, and DIP joints (including synovitis, bone oedema, STOe, flexor tenosynovitis, PTI, plantar/volar plate enhancement, collateral ligament enhancement, and erosions). Correlations between the MRI score and clinical scores were poor [45], as in other studies about synovitis. Later, the PsA Magnetic Resonance Image Score (PsAMRIS) was developed to evaluate inflammatory and destructive change in hand PsA [56], but scoring reliability was low for periarticular inflammation [57].

## 6. Correlation between Imaging and Clinical Parameters

Dactylitis may occur as an acute and tender “hot” dactylitis or as a chronic and non-tender “cold” dactylitis [4,58]. The systematic symptoms in dactylitis have been curiously neglected in the last few years [49].

A few studies have investigated the pathophysiology specific to tender dactylitis [35,45]. In one study, tender, dactylitic toes were assessed with fast spin echo-T2-weighted sequences. All 12 dactylitic toes presented with flexor tenosynovitis (mild-to-moderate fluid collection in the synovial sheaths of the flexor digitorum brevis and longus), all but one of the dactylitic toes presented with subcutaneous oedema, and joint effusion was described in more than half [35]. Healy et al. analyzed the relationship between clinical and MRI changes in 17 PsA patients (including 4 hands and 17 feet) [45]; they found that tender dactylitis had more STOe and synovitis than non-tender forms, although this association was not strong. Bone oedema was observed mostly in tender digits, ranging from periarticular involvement to oedema along the whole of the phalanx, while erosions were uncommon. None of the MRI abnormalities demonstrated a significant relationship with the clinical indices of dactylitis (including LDI).

The link between sonographic features and symptoms of the digit was first evaluated in two recent cross-sectional studies on hand dactylitis. The authors found that pain and tenderness at a digit were positively associated with the sonographic detection of flexor tenosynovitis (both in GS and PD) and STOe (both in GS and PD) [8], though inversely associated with joint synovitis (both in GS and PD). Indeed, flexor tenosynovitis and STOe were sonographic features more frequently found in hot dactylitis, while joint synovitis was the foremost sonographic feature in cold dactylitis [9].

Another cross-sectional study on 91 dactylitis cases showed that fingers with a LDI-b score above the median (12) had a significantly higher prevalence of flexor tenosynovitis and GS STOe [38]. A longitudinal study on 83 affected digits highlighted that a clinical response to treatment at 3-month follow-up also showed significant improvement in flexor tenosynovitis and STOe [59].

**Figure 2:**
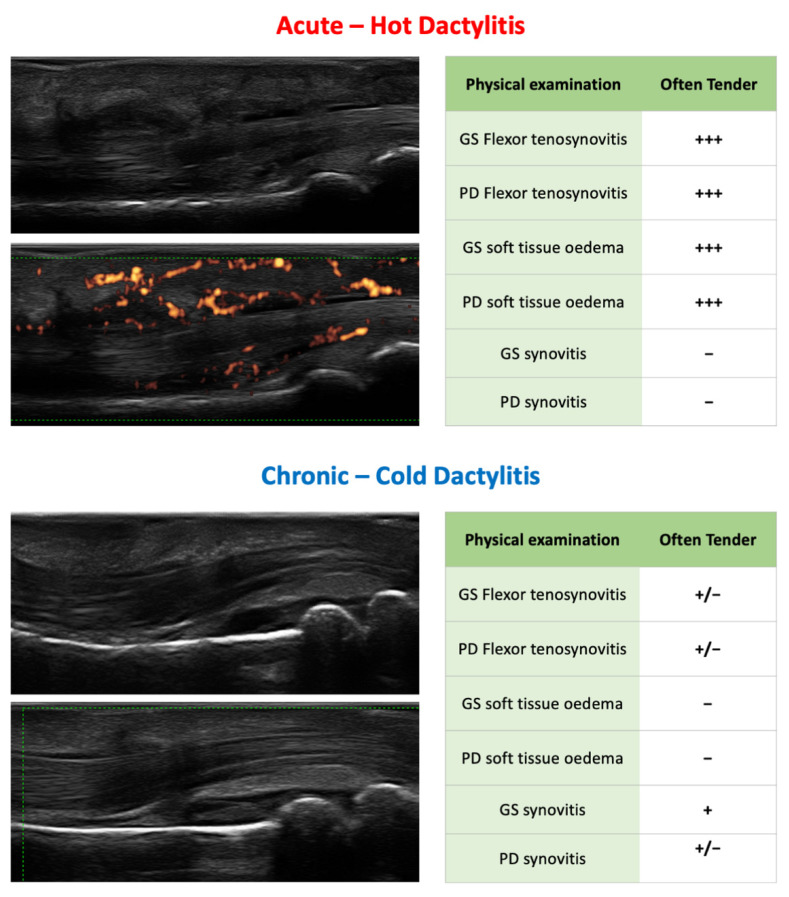
This figure summarizes the clinical and ultrasound characteristics of acute/hot and chronic/cold forms of dactylitis: Flexor tenosynovitis and soft tissue oedema are predominant in early cases, whereas synovitis is more frequent in the chronic form. Similarly, local symptoms are linked with extracapsular lesions, particularly flexor tenosynovitis and soft tissue oedema; joint synovitis does not seem to correlate with pain.

All these data suggest that local symptoms in active dactylitis are linked with extracapsular lesions, particularly flexor tenosynovitis and STOe, highlighting the central role of extra-articular structures in the genesis of digit pain. The sonographic evidence of joint synovitis does not seem to correlate with pain in the digit; this is not surprising since other rheumatic diseases demonstrate this same quality (Figure 2) [60,61,62].

**Figure 3:**
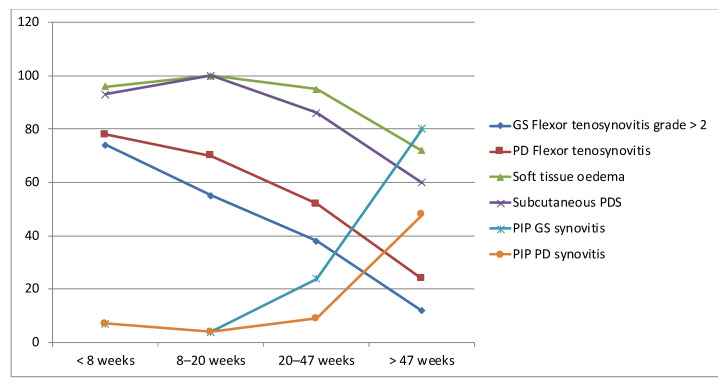
Prevalence of some US abnormalities after splitting cases into quartiles based on dactylitis duration: US: ultrasound; GS: greyscale; PD: power Doppler; PDS: subcutaneous PD signal; PIP: proximal interphalangeal. Figure reproduced from the article: Girolimetto et al. J Rheumatol. February 2020;47(2):227–233.

Some studies analyzed the correlation between the duration of dactylitis and US lesions. In a cross-sectional study of 48 dactylitic hands, patients with dactylitis less than 24 weeks had a significantly higher prevalence of a GS flexor tenosynovitis (grade > 2), PD flexor tenosynovitis, and GS and PD STOe [8]. On the other hand, synovitis was more frequent in patients with a dactylitis duration longer than 24 weeks. A subsequent study reported similar results in a larger series that included 100 affected fingers; Figure 3 reported the prevalence of flexor tenosynovitis, STOe and synovitis after splitting cases into quartiles based on dactylitis duration [39].

**Figure 4:**
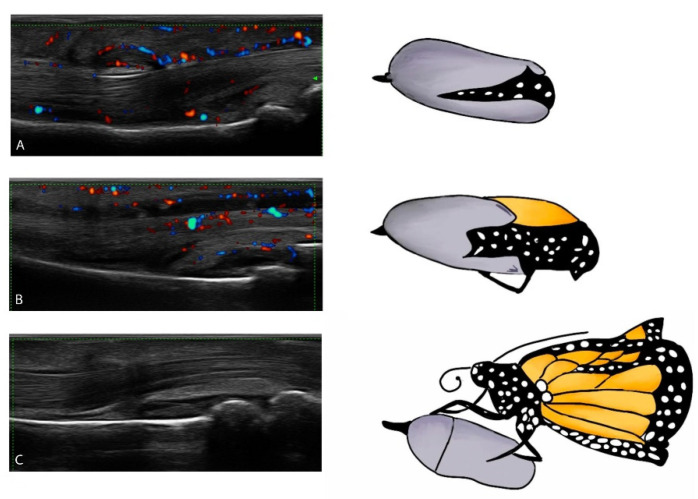
Evolution of dactylitis. Inflammation initially compromises the extracapsular structures and subsequently extends to the joint structures. The US lesions underlying dactylitis change over time, like evolving from a chrysalis to a butterfly. In particular: a) in the early phase flexor tenosynovitis and soft tissue oedema are prevalent. Joint synovitis is often absent; b) in the intermediate phase, both flexor tenosynovitis and synovitis may be present; c) in the late-chronic phase, joint synovitis is prevalent while flexor tenosynovitis is often absent or present but of minimal degree.

These findings demonstrated that flexor tenosynovitis and soft tissue oedema are predominant in early cases, whereas synovitis is more frequent in the chronic form (Figure 2). These data, along with those from MRI studies [19,63], suggest a pivotal function of the flexor tendon sheath, accessory pulleys, and extracapsular soft tissues in the pathogenesis of dactylitis. It seems that the inflammation initially compromises the extracapsular structures and subsequently extends to the joint structures (Figure 4). Our hypothesis is supported by a study which showed that tendons are the earliest structures affected by inflammation in a murine model of inflammatory arthritis [64].

## 7. US-Guided Steroid Injection in PsA Dactylitis

Traditionally, clinicians have used local corticosteroid (CST) injections to treat dactylitis. However, the recent EULAR recommendations suggest treating dactylitis similar to inflammatory polyarthritis [65]. According to these guidelines, the first-line treatment for dactylitis is conventional DMARDs, such as methotrexate, leflunomide, or sulfasalazine; patients not achieving improvement after 3 months are recommended to start biologic DMARDs. Interestingly, NSAIDs and local CST injections remained the first choice to treat enthesitis.

Nevertheless, a recent study explored the usefulness of CST injections to treat dactylitis in a multicenter prospective study. In this study, 38 dactylitic fingers were treated with local CST injection, and 35 were treated systemically. A clinically meaningful improvement in pain and functional impairment was observed at 1- and 3-month follow-up in patients treated with CST injection compared to systemic treatment, supporting the idea that CST injection could still be an effective and safe first-line therapy for psoriatic dactylitis. Even without using US-guided CST injections, the authors did not observe any injection-related side effects, such as subcutaneous fat tissue atrophy, skin hypopigmentation, tendon rupture, or infection [66].

Given that tenosynovitis is the crucial and more frequent lesion in hot dactylitis, US-guided CST injections could more easily identify and target the tenosynovial sheath. Gutierrez et al. demonstrated that US-guided CST injection for tenosynovitis (at multiple body locations) was superior to conventional blind injection; US guidance reduced injection pain and increased functional improvement [67]. Some RA studies also highlighted the efficacy of US-guided peritendinous CTS injections to manage patient symptoms [68,69].

Blind injections of CTS into the tendon sheath are fast and easy to perform; for this reason, they remain popular in clinical practice. However, the emerging evidence of the superiority of US-guided tendon sheath injections over blind injections is difficult to ignore [67].

Though studies comparing US-guided injections to those without US guidance are limited in dactylitis, we are confident that US could reduce errors, especially in learners who must correctly identify the anatomy to avoid injecting the extra-tenosynovial tissue or inflamed tendons (risking tendon rupture). For expert clinicians, blinded CST injections remain the first option to treat dactylitis.

## 8. Conclusions

Dactylitis is considered a characteristic feature of PsA, and its diagnosis is based on the clinical examination. The inflammation in dactylitis involves multiple tissues, such as entheses, peri-tendinous soft tissue, tenosynovium, articular synovitium, bone and periosteum. The inflammation is more evident in the vascular tissue surrounding avascular structures (such as tendons, entheses and pulleys) [10].

Imaging such as ultrasound and MRI can play a role in the diagnostic and follow-up process, especially in doubtful cases, where the clinical examination might be not completely reliable (e.g., high BMI or early arthritis presenting mild digit inflammation) [43]. Imaging can visualize the underlying inflammatory mechanism in different clinical manifestations among various disease duration. In dactylitis with short duration, soft tissue oedema and tenosynovitis are the prevalent lesions compared to synovitis. The opposite happens in dactylitis with longer duration, in which synovitis is more prevalent than tenosynovitis and soft tissue oedema, supporting the idea that articular inflammation in PsA may initiate outside the synovium and then spreads into the synovial tissue.

Furthermore, imaging techniques may also be useful in clinical trials, monitoring the response to treatment of dactylitis and differentiating the response of each dactylitic elementary component.

## Data Availability

Data sharing not applicable.
